# The defensive playing style was associated with match-running performance and team success in the FIFA Women's 2023 World Cup

**DOI:** 10.3389/fspor.2026.1693238

**Published:** 2026-04-10

**Authors:** Luís Branquinho, Elias de França, Adriano Titton, Francisco Tomás González-Fernández, José E. Teixeira, Pedro Forte, Carlos E. Lopes Verardi, Vinicius Barroso Hirota, Ricardo Ferraz, Israel Teoldo, Ronaldo Vagner Thomatieli-Santos

**Affiliations:** 1 Biosciences Scholl of Elvas, Polytechnic University of Portalegre, Elvas, Portugal; 2 Life Quality Research Center (LQRC-CIEQV), Santarem, Portugal; 3 Research Center in Sports Sciences, Health Sciences and Human Development, Covilhã, Portugal; 4 Centro de Investigação do Instituto Superior de Ciência Educativas (CI-ISCE), Penafilel, Portugal; 5 Interdisciplinar Graduate Program in Health Sciences, Universidade Federal de São Paulo, Santos, Brazil; 6 Human Movement Laboratory, São Judas University, São Paulo, Brazil; 7 São Paulo Futebol Clube, São Paulo, Brazil; 8 Centre of Research and Studies in Soccer (NUPEF), Universidade Federal de Viçosa, Viçosa, São Paulo, Brazil; 9 Department of Physical Education and Sports, Faculty of Sport Sciences, Sport and Health University Research Institute (iMUDS), University of Granada, Granada, Spain; 10 Polytechnic Institute of Bragança, Bragança, Portugal; 11 Polytechnic Institute of Guarda, Guarda, Portugal; 12 SPRINT—Sport Physical Activity and Health Research & Innovation Center, Rio Maior, Portugal; 13 Department of Sport Sciences, Polytechnic Institute of Cávado e Ave, Guimarães, Portugal; 14 LiveWell—Research Centre for Active Living and Wellbeing, Bragança, Portugal; 15 Sports Department, Higher Institute of Educational Sciences of the Douro, Penafiel, Portugal; 16 Faculdade de Ciências, Curso de Educação/física, Universidade de São Paulo, Bauru, São Paulo, Brazil; 17 Technological Graduation in Sports and Leisure Management, FATEC of Sports, São Paulo, Brazil; 18 Sports Sciences Department, University of Beira Interior, Covilhã, Portugal; 19 Graduate Program in Psychobiology, Universidade Federal de São Paulo, São Paulo, Brazil

**Keywords:** playing style, soccer running performance, tactical defense, technical defense, women soccer

## Abstract

**Introduction:**

Currently, there are no data in the women's soccer literature about playing style without ball possession and its impact on match-running performance or competitive success. The main objective of this study is to verify whether there was a relationship between match-running performance, team success, and playing style without ball possession in the FIFA Women's 2023 World Cup.

**Methods:**

Twenty-one variables related to tactical and technical behavior without ball possession and match-running performance were collected from 64 matches (from 32 national teams) using a multicamera optical tracking system. A machine-learning approach was used to describe the playing style profile and its relationship with match-running performance. A chi-square test was then used to verify the association between playing style and team success.

**Results:**

The data indicated that teams that adopted tactical marking behavior with high-press, mid-block, and recovery saw increased match-running demands. On the other hand, technical actions such as direct pressure and tackles saved running demands. The machine-learning analysis identified two playing styles without ball possession: (1) “low- and mid-block and low-press” and (2) “high-block and high- and mid-press” playing style without ball possession. The “high-block and high- and mid-press” playing style entailed high-intensity running and was associated with team success in the 2023 World Cup, while the “low- and mid-block and low-press” playing style was associated with unsuccessful teams.

**Discussion and conclusions:**

Playing style without ball possession significantly impacted match-running performance and team success in the 2023 World Cup. Specifically, successful teams adopted a “high-block and high- and mid-press” playing style. Such a playing style adopts a high-press, recovery, defensive transition, counter-press, and high-block profile, which demands more running at high intensity.

## 1 Introduction

Over the last few years, there has been a gradual growth in interest related to women's soccer, largely due to the strong investment made by various organizations (1). As a consequence, there has been an exponential growth in the number of practitioners and spectators, as well as a notable increase in research specifically related to women's soccer (2).

Nevertheless, until now, the most investigated topics have been related to causes and prevention of injuries (3, 4) and strength and physical conditioning (2, 5, 6). This is indeed an alarming indicator as a knowledge of the relationships between different domains (i.e., physical, technical, and tactical) can be fundamental to the broad understanding of the demands that women soccer players may face during games and competitions.

It is now widely accepted that soccer is characterized by high physical demands (7, 8). Women's running in soccer alternates between periods of high intensity (i.e., sprinting above 25 km/h) and periods of moderate to low intensity (i.e., walking at 0.7–7 km/h) and covers distances between 9 and 11 km during a match (7, 9–11). To clarify and understand these demands in detail, match-running performance has been one of the most used factors (9, 12–14). Recent evidence demonstrates the existence of significant differences in the time spent at low and high intensity, which may be due to the influence of several factors associated with the match (7, 9, 15, 16). The distance covered at high intensity is considerably smaller (17, 18). However, the actions carried out during these periods occur at key moments in the game (19, 20) and represent approximately 12% of the total distance covered (21). These data are certainly useful for clarifying the physical demands of soccer but should not be interpreted in isolation (15, 22). For instance, data regarding the influence of tactical behavior on match-running performance adopted in women's soccer are lacking. The only study using female participants that addressed this theme identified that offensive tactical choices affect the distance covered (23). For example, higher ball possession and progression can prevent athletes from covering greater distances. On the other hand, teams that choose to exchange more passes and make more breaks in the defensive lines cover more distance than their counterparts. Therefore, information regarding which tactical behavior affects/demands higher running intensity could provide valuable information for coaches and staff.

An analysis of playing style in soccer is recent, and to the author's knowledge, there are no data in the literature addressing this issue in women's soccer. Playing style is the result of the tactical options chosen by a team (24). Analyzing playing style is essential because it has been identified in male players that there are successful playing styles with (25–27) and without ball possession (28, 29). For instance, during a game, a team can adopt different types of collective tactical behaviors (i.e., playing style) in different phases of play (i.e., with or without ball possession) (30). In fact, playing style assumes a preponderant role for players to achieve excellent performances, as it must be understood as the correct management of the playing space occupied by the players in relation to different domains (i.e., movement, positioning, and displacement) (31, 32). Therefore, the possibility of a strong relationship between the movement of players on the field (i.e., playing style) and match-running performance indicators seems feasible, and this could have an influence on match-running performance indicators throughout a competition (e.g., FIFA Women's World Cup); however, this remains to be clarified. To date, numerous investigations have explored the relationship between match-running performance and success in different components of the game (1, 7, 9, 13, 22). Understanding how physical performance, expressed through metrics (i.e., distance covered, speed, and sprints), is related to specific game tasks in different phases (i.e., playing style with and without ball possession) can offer valuable information for the development of more effective tactical strategies. In fact, it seems important to clarify in women's soccer how the collective tactical behavior (playing style) adopted by a team in different game phases affects the match-running performance throughout a game and/or competition.

Therefore, the main objective of this study is to verify whether there was a relationship between match-running performance and behavioral patterns without ball possession (playing style) in the teams that participated in the FIFA Women's 2023 World Cup. The second aim of this study is to verify whether playing style without ball possession was also associated with team success or failure in that World Cup.

## 2 Methods

### 2.1 Participants

The sample consisted of 32 national teams that competed in the 2023 World Cup, which included 64 games and produced a total of 128 distinct datasets (one per team in each game played). Of these 128 datasets, eight were excluded because they were from games with extra time; also, ten datasets from games with red cards were excluded (thus, we used 110 data from 55 games; see Supplementary Table S1). The teams played anywhere between three and seven games, depending on their performance in the competition. In each game, a total of 21 variables related to the game were extracted. No ethical approval was required for this study, as the data of this study are freely available (33).

### 2.2 Experimental approach to the problem

This is an observational and exploratory study of the 9th edition of the Women's World Cup. Data related to the 64 matches were provided by FIFA (33) as a PDF postmatch summary and all variables were collected and organized in an Excel (Microsoft, v.365, 2024) spreadsheet. The data were collected by FIFA using a multicamera optical tracking system (TRACAB Gen5, ChyronHego). The validity and accuracy of data collection for running performance in soccer by TRACAB Gen5 were provided previously (34). After the data were captured, the tactical variables were processed using match analysis software (Viz Libero software, Bergen, Norway). The validity and reproducibility of the tactical variables presented in this study (Table 1) were provided by Ju et al. (35).

**Table 1:** The concepts of the two categories and the theoretical foundation of all respective variables.

Phases of the game or behavior without possession of the ball	Phases of the game or technical/ tactical actions	Marking profile without possession of the ball	Type of actions	Description
Phase of play, out of ball possession	Out of ball possession (% of time spent in action without ball possession)	Aggressiveness profile of the marking	High-press	In the opponent's field, players pressure the opponent with ball possession with attempts to regain ball possession while teammates mark their passing options.
			Mid-press	Team moves as a unit preventing the opponent's progression to the last third of the field.
			Low-press	Team moves as a unit but applies passive pressure allowing the opponent to reach the last third of the field.
		Marking block height	High-block	A compact team in the offensive field to keep the ball in the opponent's defensive field. The high block tries to win the ball back in the final third of the field in the opponent's buildup phase.
			Mid-block	Compact team in the middle third of the field.
			Low-block	Compact team in the defensive third of the field, preventing penetration in the area.
		Tactical approach after losing possession of the ball	Recovery	Run toward their defensive pitch after losing possession of the ball.
			Defensive transition	Recovery into their defensive shape.
			Counter-press	Aggressive pressure on the opponent after losing ball possession.
Behavior out of ball possession	Number of technical actions intended to regain ball possession	Forced turnovers		Recovered ball due to applied pressure.
		Possession regained		Due to different ways (interception, a poor pass from an opposing player, tackle, or second ball).
		Interceptions		Intercept the ball with the intention of winning possession during a pass, long pass, or cross from the opponent team.
		Tackles		An attempt by a player to recover the ball's possession using face-to-face (sliding, pushing, or blocking) confrontation.
	Number of tactical actions intended to regain ball possession	Total pressures		The total number of actions that the athlete approaches the opponent to reduce their attacking options or try to win ball possession.
		Direct pressures		Explicit and aggressively defensive action on an opposing player to compete for ball possession.
		Average Pressure duration		Average time spent pressuring opponent teams.
		Ball recovery time		The time it takes for a team to regain possession of the ball after losing possession.
		Pushing on into pressing		Pushing the opponent who will receive the ball.
		Pushing on		A player attempts to close down the space between themselves and an opposition player when the opposition player does not have the ball.
		Pressing direction inside		A player makes a deliberate attempt to force the opposition player with the ball toward the center of the pitch.
		Pressing direction outside		A player makes a deliberate attempt to force the opposition player with the ball away from the center of the pitch.

Variable descriptions adapted from de França et al. (39).

### 2.3 Procedure

To characterize playing style without ball possession, 21 variables were manually categorized into two broad categories: (1) phase of play, out of ball possession (nine variables); and (2) behavior out of possession (13 variables) (see Table 1). These two categories were created to assess the two moments of play in the game phase without ball possession: the “transition from attack to defense” and “established defense” (24).

To identify whether playing style patterns without ball possession affect total and high-intensity running covered during matches, we extracted the total team running distance (km) and team high-intensity distance (>20 km/h) covered.

As a contextual variable, we included ball possession in the playing style assessment. Thus, it is possible to identify which play style without ball possession is associated with the profile of the team during ball possession. Also, the team success in the 2023 World Cup was classified in quartiles according to the teams’ respective 2023 World Cup final classifications for the tournament (eight teams with their respective performance cases, described in Supplementary Figure S1).

### 2.4 Statistical analysis

Data were presented as mean and standard deviation (SD) [or, if indicated, confidence interval (CI) 95%]. To determine whether actions performed without ball possession influenced running performance, a mixed linear model was applied, with the total running distance (km) and high-intensity running distance as dependent variables. Variables describing actions without ball possession were entered into the model as covariates. As data from the same team were used several times, team identification was used as random effects (intercept model). A decision tree regression model was created to establish the amount of action that could explain the match-running performance variation. The decision tree regression model was designed using the CHAID method for spontaneous growth. The nodes (leaves) of the decision tree regression were pruned to compare at least 10 samples in each node. Also, the 21 variables from game phases without ball possession (described in Table 1), ball possession, and match-running performance were standardized to z-score, and the K-means cluster analysis was employed to identify the defensive playing style. This analysis was also used to identify the most meaningful cluster solution (between two and five clusters). Data visualization in hierarchical clusters and high silhouette scores identified that two clusters were the best solution. The K-means algorithm used Euclidean distance to compute distances and defined 100 iterations to compute the cluster centroids. Differences between clusters were verified using ANOVA one-way followed by Duncan *post hoc*. After cluster creation the chi-square analysis was used to identify the association between clusters with 2023 World Cup final ranking. All analyses were performed using the statistical package IBM SPSS Statistics v.26.0, with significance set at ≤0.05.

## 3 Results

Tables 2, 3 describe the profile of the phase of play without ball possession intended to regain ball possession. Table 2 describes the percentage of how teams spend their time without ball possession. For instance, in the high-press variable presented in Table 2, a team could spend 10% of their time without ball possession performing a high-press in the opposition defensive half, while other teams performed no high-press. Table 3 describes the number of activities (and success) to recover the ball. A large variation could be seen for several variables (in both Tables 2, 3), indicating different team behavior profiles without ball possession during the matches.

**Table 2:** Descriptive data of the phases of play out of ball possession behavior and the total distance covered during matches in the 2023 World Cup.

Variables	Mean	SD	Min.	Max.
High-press (%)	3.47	1.9	0.0	10.0
Mid-press (%)	5.65	2.1	1.0	12.0
Low-press (%)	1.0	0.6	0.0	3.0
High-block (%)	4.8	3.1	0.0	14.0
Mid-block (%)	18.3	9.3	4.0	52.0
Low-block (%)	15.3	10.4	1.0	55.0
Recovery (%)	4.22	1.9	1.0	10.0
Defensive transition (%)	21.5	6.6	9.0	37.0
Counter-press (%)	15.4	4.5	7.0	26.0
Total distance covered (km)	108.2	6.0	91.5	123.0
High-speed distance covered (km)	4.9	0.6	3.3	6.7
Ball possession^a^ (%)	42.5	13.3	18.8	68.2

The percentage of time in defensive behavior is relative to the total time without ball possession (not the total time of the match). Thus, the sum of variables related to defensive behavior is approximately equal to 100% of the total time without ball possession.

^a^ The ball possession percentage reported here is relative to the time of the match. Also, the values are subtracted from the time in which the ball is contested.

**Table 3:** Descriptive data of actions without ball possession during matches in the 2023 World Cup.

Variables		Mean	SD	Min.	Max.
Defensive actions	Forced turnovers (N)	82.55	12.96	53	115
	Possession regained (N)	56.29	8.60	38	80
	Interceptions (N)	8.45	3.52	3	19
	Tackles (N)	37.86	10.98	12	73
Defensive pressure	Total pressures (N)	226.75	74.51	96	460
	Direct pressures (N)	60.12	16.03	27	107
	Average pressure duration (s)	1.47	0.16	1.01	1.96
	Ball recovery time (s)	9.64	3.14	4.57	21.57
	Pushing on into pressing (N)	85.68	36.36	20	211
	Pushing on (N)	193.50	76.64	6.65	404
	Pressing direction inside (N)	35.45	13.28	12	86
	Pressing direction outside (N)	117.70	46.22	38	265

Adopting a tactical profile without ball possession, such as high-press, mid- and low-block, and recovery positively affects the total running distance covered (see Table 4). Also, adopting high-press and recovery positively influences the high-speed distance covered, while adopting low-block negatively impacts high-speed distance covered (see Table 5).

**Table 4:** Mixed linear model for the total distance covered and time spent in defensive behaviors.

Variables	Parameter estimates	95% Confidence interval		*P*-values
		Lower bound	Upper bound	
Intercept (km)	91.25	78	104.49	**<0**.**001**
High-press (%)	0.7	0.05	1.35	**0**.**005**
Mid-press (%)	−0.25	−0.74	0.23	0.3
Low-press (%)	−1.5	−2.97	−0.03	0.19
High-block (%)	0.14	−0.24	0.52	0.47
Mid-block (%)	0.47	0.28	0.65	**<0**.**001**
Low-block (%)	0.22	0.03	0.42	**0**.**02**
Recovery (%)	0.7	0.18	1.23	**<0**.**01**
Defensive transition (%)				
Counter press (%)	0.38	−0.04	0.79	0.11
Ball possession	0.13	0.01	0.26	**0**.**03**

Defensive transition (%) was removed due to high collinearity.

Values ​​in bold represent statistical significance at *p* < 0.05 level.

**Table 5:** Mixed linear model for high-speed distance covered and the time spent in defensive behaviors.

Variables	Parameter estimates	95% Confidence interval		*P*-values
		Lower bound	Upper bound	
Intercept (km)	4.04	2.51	5.56	**<0**.**001**
High-press (%)	0.08	−0.00007	0.15	**0**.**05**
Mid-press (%)	−0.01	−0.07	0.04	0.94
Low-press (%)	0.15	−0.02	0.32	0.07
High-block (%)	0.01	−0.03	0.06	0.58
Mid-block (%)	0.01	−0.01	0.03	0.48
Low-block (%)	−0.02	−0.04	0.0003	**0**.**05**
Recovery (%)	0.13	0.07	0.19	**<0**.**001**
Defensive transition (%)				
Counter press (%)	−0.01	−0.05	0.04	0.76
Ball possession	−0.01	−0.2	0.001	0.08

Values ​​in bold represent statistical significance at *p* < 0.05 level.

**Figure 1:**
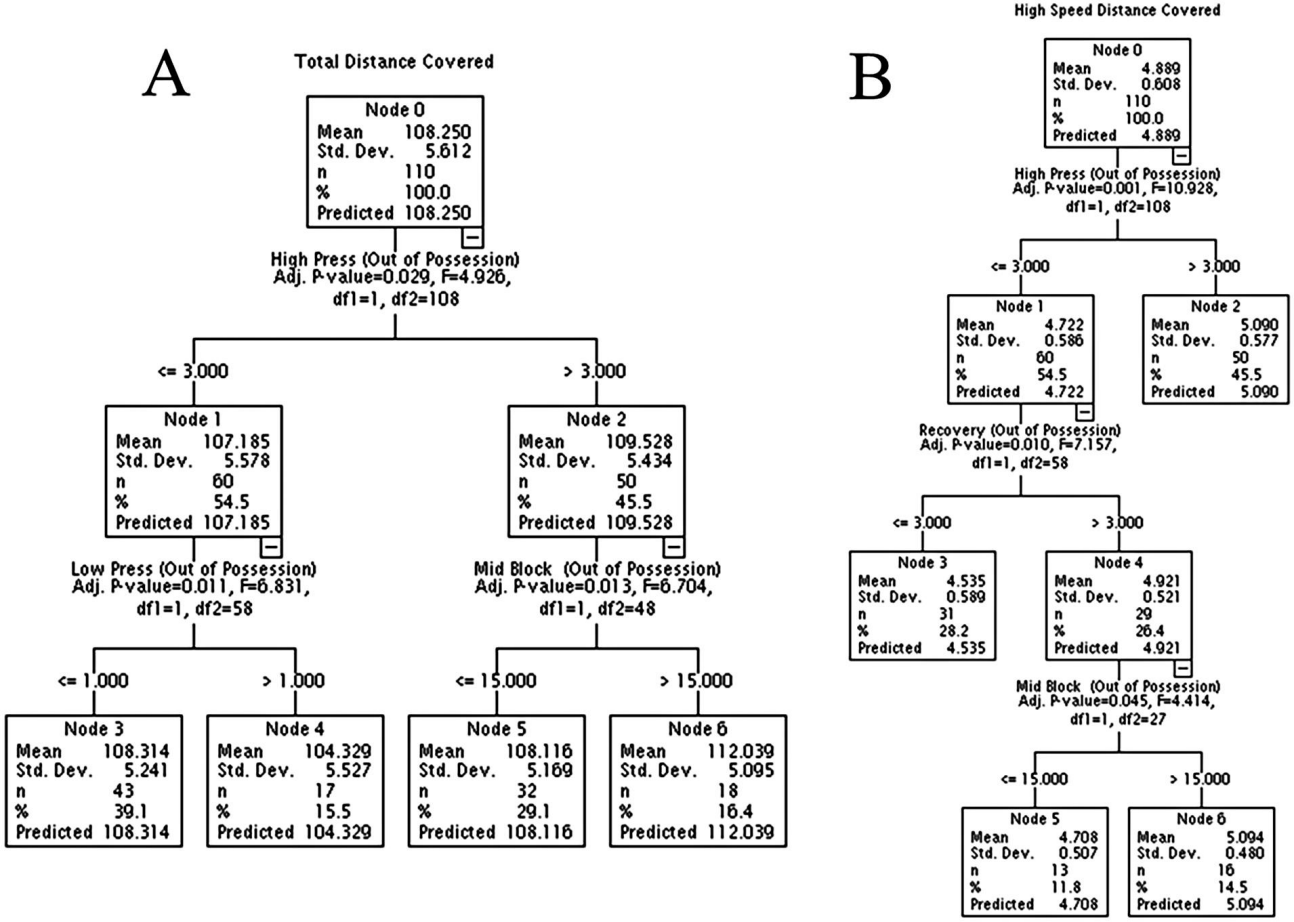
Decision tree for running performance and marking profile. **(A)** shows the association between total distance and marking profile. **(B)** shows the association between high-speed distance and marking profile.

The decision tree (Figure 1) identified performance cases that adopted >3% of high-press behaviors, which induced significant increases in the total and high-intensity distances covered. Nodes 3 and 4 (Figure 1A) identify that teams that spent lower time in high-press activity (<3%) and execute lower-press activity (>1%; Figure 1A) decrease the total distance covered. This model explains 16% of the total distance variation. On the other hand, teams with high activity in high-press (Figure 1A), which also adopt high activity in middle blocks (Nodes 5 and 6), increase the total running distance.

In Figure 1B, the high-speed distance is also positively affected by the adoption of high-press, recovery, and mid-block playing style. This model explains 19% of the high-speed distance variation.

**Figure 2:**
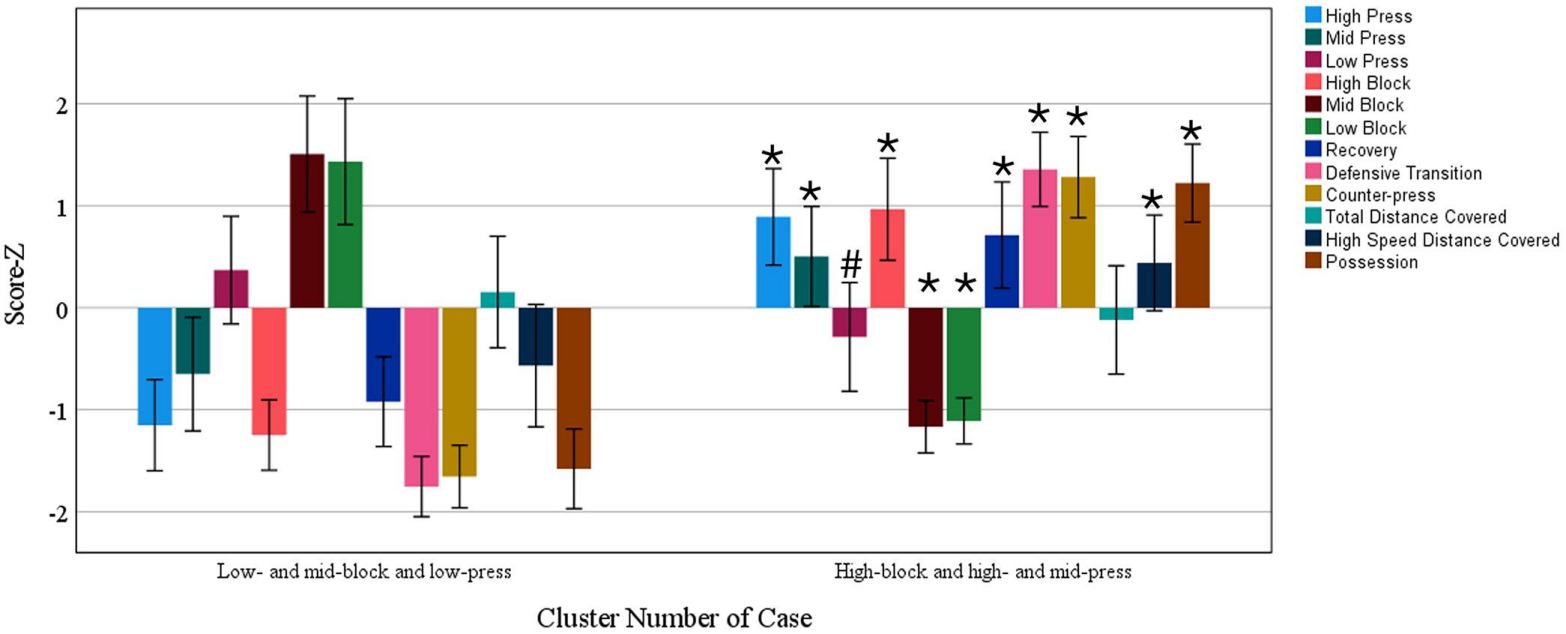
Clusters of out-of-possession activity (i.e., playing styles) of women in the 2023 World Cup. #, *P* = 0.08 when compared with the “low- and mid-block and low-press” cluster. *, *P* < 0.05 when compared with the “low- and mid-block and low-press” cluster.

Based on the higher silhouette scores (0.7, good), two clusters were created identifying the defensive play style used during the 2023 World Cup. Figure 2 shows two different playing styles with 48 and 62 performance cases for “low- and mid-block and low-press” clusters, and “high-block and high- and mid-press” defensive clusters, respectively. The “low- and mid-block and low-press” cluster differs significantly from the “high-block and high- and mid-press” cluster in all variables except for the total distance covered. Specifically, the “high-block and high- and mid-press” cluster has a notable characteristic of operating in a high-block, high-pressure, recovery, defensive transition, and counter-press tactical behavior. We included ball possessions and the total distance covered to create clusters. Thus, it was possible to identify which play style without ball possession was associated with the profile of team ball possession and running distance. We found that “high-block and high- and mid-press” had high possession and high-speed activity.

**Figure 3:**
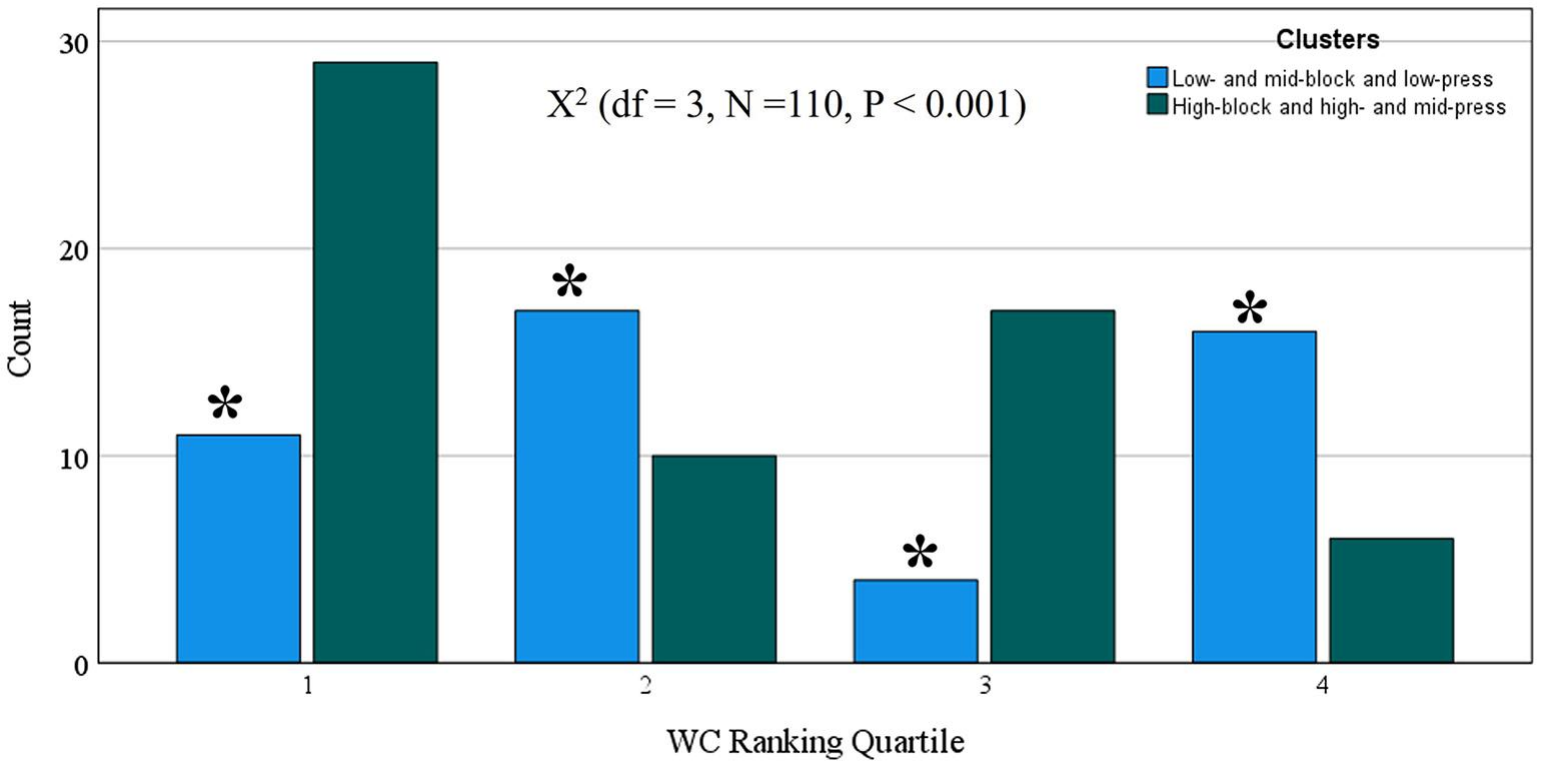
Association between clusters of playing styles without ball possession and quartile final classification in the 2023 World Cup. *, denote statistical difference between play style in the same quartile.

To investigate whether the clusters (presented in Figure 2) were associated with team success in the 2023 World Cup, we divided the 32 teams into four quartiles according to their final classification in the competition (see Supplementary Tables S1 and S2). In Figure 3, we performed the chi-square test and identified a significant association between the clusters and the team's success in the 2023 World Cup (*P* < 0.001), with the “ high-block and high- and mid-press” cluster playing style being the most used by teams in the first quartile and the least used by teams in the fourth quartile.

In the defensive action activities, performing tackles was negatively associated with the total distance covered (Table 6). Also, in defensive pressure activities, direct pressure was negatively associated with the total distance covered. However, teams with high pushing on into pressing and pressing direction outside were positively associated with the total distance covered (Table 6).

**Table 6:** Mixed linear model for the total distance covered and defensive actions.

Variables		Parameter estimates	95% Confidence interval		*P*-values
			Lower bound	Upper bound	
	Intercept (km)	111.77	104.12	119.41	<0.001
Defensive actions	Forced turnovers (N)	0.06	−0.03	0.15	0.22
	Possession regained (N)	−0.02	−0.17	0.13	0.77
	Interceptions (N)	−0.05	−0.37	0.27	0.75
	Tackles (N)	−0.17	−0.27	−0.07	<0.001
	Intercept (km)	94.89	81.32	108.45	<0.000
Defensive pressure	Total pressures (N)				
	Direct pressures (N)	−0.09	−0.17	−0.01	0.03
	Average pressure duration (s)	−2.56	−8.59	3.47	0.40
	Ball recovery time (s)	0.39	−0.23	1.02	0.21
	Pushing on into pressing				
	Pushing on (N)	−0.01	−0.14	0.09	0.30
	Pressing direction inside (N)	−0.02	0.04	0.15	0.69
	Pressing direction outside (N)	0.09	0.13	0.39	<0.00
	Ball possession	0.26	0.13	0.39	<0.001

Total pressures and pushing on into pressing were removed due to high collinearity.

Only ball recovery time showed a negative trend associated with high-speed distance covered in defensive pressure (Table 7).

**Table 7:** Mixed linear model for high-speed distance covered and defensive actions and pressure.

Variables		Parameter estimates	95% Confidence interval		*P*-values
			Lower bound	Upper bound	
	Intercept (km)	5.39	5.39	4.85	**<0**.**001**
Defensive actions	Forced turnovers (N)	0.02	0.02	0.02	0.15
	Possession regained (N)	0.01	−0.004	0.03	0.16
	Interceptions (N)	1.14	1.14	0.01	0.21
	Tackles (N)	0.02	0.02	0.01	0.36
	Ball possession	0.009	0.001	0.018	
Defensive pressure	Intercept (km)	5.07	3.70	5.39	**<0**.**001**
	Total pressures (N)				
	Direct pressures (N)	−0.001	−0.01	0.01	0.99
	Average pressure duration (s)	0.11	−0.56	0.79	0.75
	Ball recovery time (s)	−0.07	−0.13	0.00	0.057
	Pushing on into pressing				
	Pushing on (N)	0.00	0.00	0.00	0.38
	Pressing direction inside (N)	−0.01	−0.02	0.00	0.11
	Pressing direction outside (N)	0.003	−0.003	0.009	0.29
	Ball possession	0.003	−0.01	0.02	0.66

Total pressures and pushing on into pressing were removed due to high collinearity.

Values ​​in bold represent statistical significance at *p* < 0.05 level.

The decision tree did not form branches to defensive actions and defensive pressure activities.

**Figure 4:**
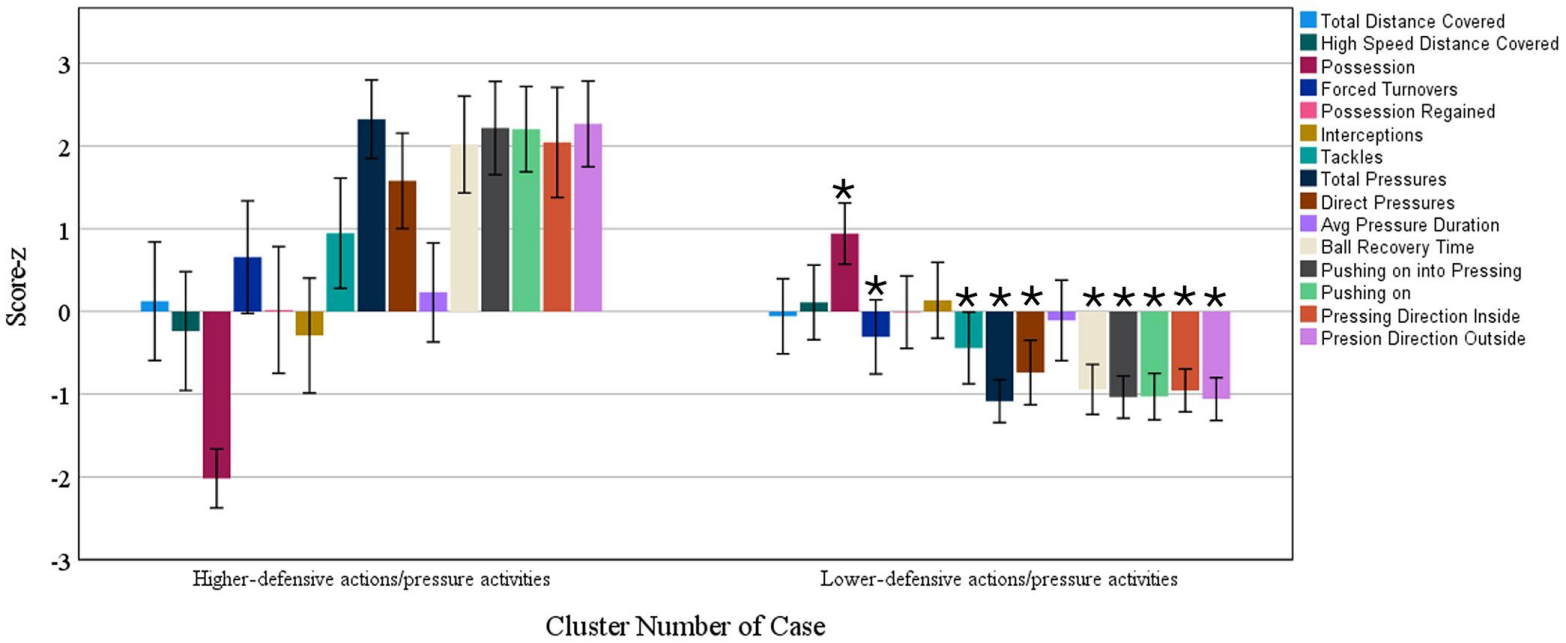
Clusters of actions without ball possession. *, *P* < 0.05 when compared with the “higher defensive actions/pressure activities” cluster.

**Figure 5:**
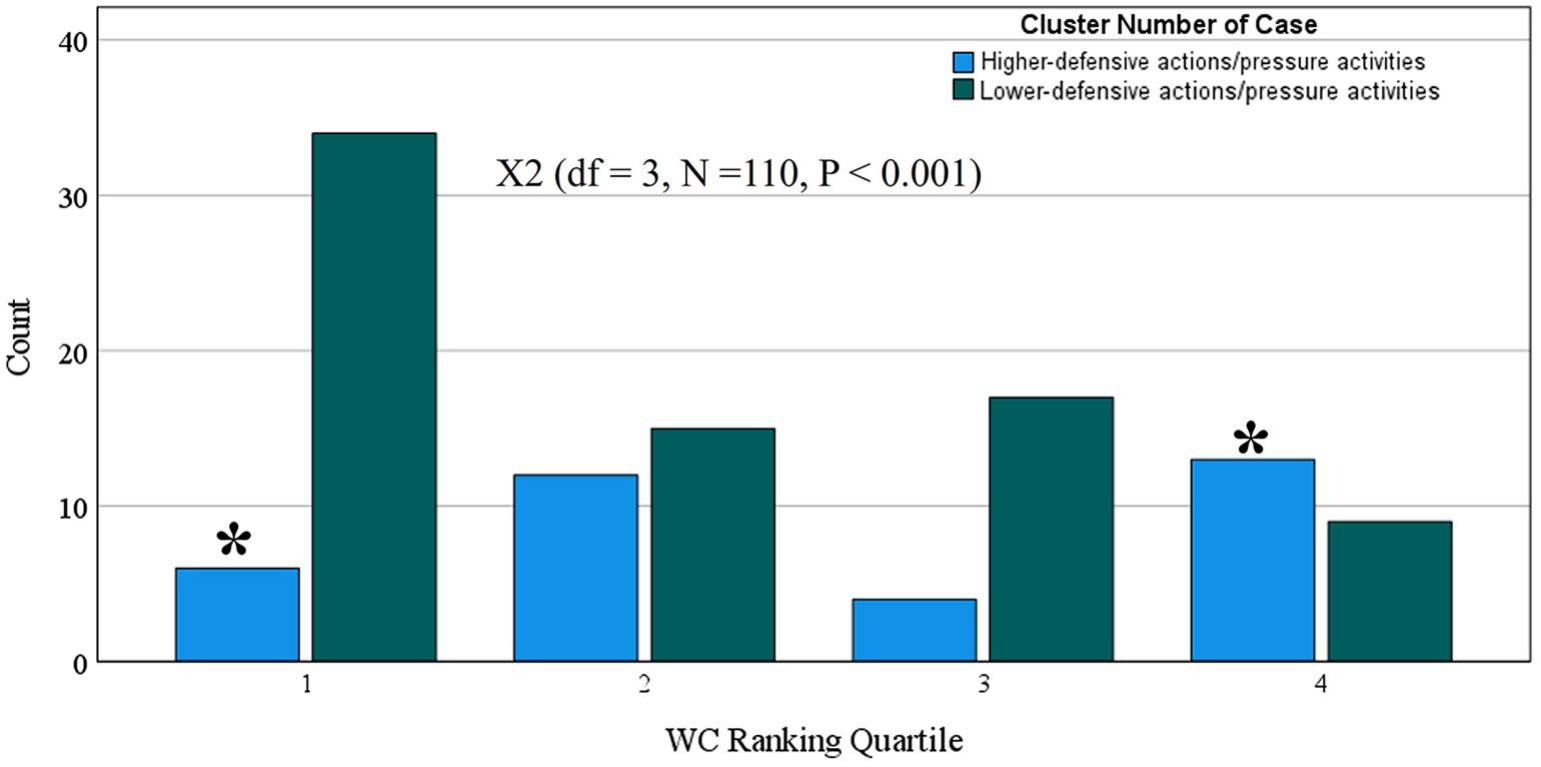
Association between clusters of actions without ball possession and quartile final classification in the 2023 World Cup. The different letters denote significant differences (*P* < 0.05) between clusters in the same quartile for the Z-test with the Bonferroni method.

In Figure 4, we present that the two different profiles of actions without ball possession (silhouette score = 4, fair) have no association with running performance; however, they were significantly associated with success in the 2023 World Cup (see Figure 5). There was a significant association between the clusters and the team's success in the 2023 World Cup (*P* < 0.001), with the “lower-defensive/pressure activities” cluster being the most used by teams in the first quartile and the least used by teams in the fourth quartile.

## 4 Discussion

The main finding of this study identified that tactical behavior (playing style without ball possession) is associated with the distance covered. Furthermore, playing style without ball possession was associated with the team's success in the 2023 World Cup. To our knowledge, no previous research has described playing style without ball possession in women's soccer.

This study shows that tactical playing style without ball possession significantly impacts the total running distance (volume) and speed (intensity). Specifically, the playing style without ball possession that adopts a high-block marking also adopts high-press, which is associated with recovery, defensive transition, and counter-press behavior; moreover, this playing style entails running a significantly high-speed distance when compared with other playing styles identified in this study. The “high-block and high- and mid-press” playing style assumes a tactical pattern that requires players to run a greater distance at a higher intensity when compared with other playing styles. Similarly, a study by Plakias et al. (36) in men's soccer showed that high-pressure teams, compared with low-pressure teams, demonstrated running higher distances combined with high-intensity running, as well as participating in high-speed events/sprints at greater frequency. The intensity of high-speed distance assessed in this study (i.e., > 20 km/h) lies within the anaerobic zone. Therefore, we hypothesize that teams that want to adopt the “high-block” playing style must train their athletes in the anaerobic speed zone.

The “low- and mid-block and low-press” style covers the same total distance as the “high-block and high- and mid-press” playing style. However, it was associated with poor performance in the 2023 World Cup; notably, it was the playing style most used by teams in the last quartile of the tournament. Furthermore, the “low- and mid-block and low-press” playing style was the least used in the first quartile, thus suggesting that adopting this style during the matches can lead to defeat. In this sense, future investigations in women's soccer verifying the contextual factors (such as game status, venue, quality of the opponent, and their interactions) that are known to affect playing style without ball possession in men's soccer (37) will be of great importance for coaches and staff.

A study (24) identified that a soccer match has five moments of play: (1) established attack, (2) transition from defense to attack, (3) set pieces, (4) transition from attack to defense, and (5) established defense. This study assesses items 4 and 5 and identifies a clearly different playing style between the “high-block and high- and mid-press” playing style (which adopts an aggressive posture to recover the ball immediately after losing the ball in the attacking field) and the “low- and mid-block and low-press” playing style (in which the team applies passive pressure in the first and second thirds of the field). Our linear mixed model (and the clustered K-means) analysis indicates that variables associated with the “high-block and high- and mid-press” playing style, such as high-press, recovery, and counter-press, play a fundamental role in the total distance covered. If the ball is lost, the adoption of the “high-block” playing style requires players to put intense pressure on the opposing team (e.g., counter-press and high-press), and they should try to recover the ball’s possession while they are still in the opposition’s defensive half. Furthermore, as the team adopts marking in the final third of the field, it implies the need to execute greater amounts of recovery. This impacts the distance covered by this playing style without ball possession. Our three analyses (linear mixed model, decision tree, and cluster K-means) also indicate that adopting the mid-block defense *per se* impacts the distance covered. Particularly, teams that spend greater than 15% of their time (using mid-block marking) without ball possession tend to run greater distances when compared with performance cases that spend ≤15% of their time indulging in mid-block behavior (Figure 1). These data are in accordance with the literature on men’s soccer, where mid-block adoption is associated with greater distances covered (36).

According to our linear mixed model analysis, defensive actions and pressures did not significantly impact the total and high-speed running distance covered in the 2023 World Cup. Thus, these data suggest that tactical variables from without ball possession phase of play (described in Figure 2) are more impactful than technical variables (described in Figure 4) in running distance. Also, our cluster analysis shows that a high number of defensive actions and pressures are associated with lower ball possession. A previous analysis (23) showed that higher ball possession was associated with many offensive actions and team success in the 2023 World Cup. In this sense, having a high number of defensive actions and pressures is a consequence of not having ball possession (see Figure 5). When creating the clusters in this study (Figure 5), ball possession was added as a contextual variable to demonstrate that performances that have a high number of defensive actions and pressures are low ball possession performances, which certainly explains the fact that defensive actions are not associated with running distance but with ball possession.

This study identified that a playing style without ball possession was associated with team success in the 2023 World Cup; this style requires athletes to cover greater high-intensity running distances. This has direct effects on physical preparation, and once successful, this playing style might require greater physical conditioning from athletes. Adopting high-block behavior requires greater participation from attacking players performing the marking task. In this sense, future analyses verifying the match-running volume and intensity, mainly by midfield and attacking players subjected to these different playing styles without ball possession, can provide more specific information for direct physical preparation.

However, this study has some limitations. First, the FIFA Women’s 2023 World Cup has characteristics that make data extrapolation difficult. For example, the factor of home advantage may exist only for the host team, and public support is not guaranteed for the other teams. Therefore, care must be taken when extrapolating data for championships in which the advantage of playing at home is real. Furthermore, the data in this study need to be validated in a league competition because the Women’s 2023 World Cup had a stage group following a knockout stage. Therefore, future studies are needed to verify whether the same playing style associated with team success seen in this study also applies to a league competition. Also, such data need to be validated in competitions involving extensive travel, which is known to affect match-running performance (38). Second, the running distance data used in this study encompass the entire game. Ideally, the analysis reported in this study should also include the distance covered without ball possession.

This study has some practical applications. The study identified different defensive playing styles in the Women’s 2023 World Cup. Thus, the analysis employed in this study can be used by scouting teams to identify defensive playing styles of opposing teams or their own team. Also, the data from this study can be used to guide tactical training to create a successful defensive playing style. The analysis from this study also indicates that a high level of physical fitness is necessary to adopt a successful defensive style of play. Moreover, coaches need to pay more attention than others because the clusters created from defensive actions and pressures (Figure 4) were significantly associated with team success in the 2023 World Cup (Figure 5). For example, the clusters that involved activities with a medium or high number of defensive actions and pressures were associated with the last quartile of the 2023 World Cup, while the cluster that had a low number of actions and pressures (including short time of ball recovery = ∼8 s) had the highest frequency in the first quartile and the lowest frequency in the fourth quartile of the 2023 World Cup ranking. These same patterns were previously identified in the 2022 World Cup (39). Thus, if the coach identifies that their team in the competition is executing actions and defensive pressures above the competition average, they need to keep in mind that this is an indicator that their team may be unsuccessful in the competition (25) and must check the quality of its defensive strategies (29).

## 5 Conclusion

This investigation demonstrates that there was a relationship between match-running performance and behavioral patterns without ball possession (playing style) in the FIFA Women’s 2023 World Cup. In addition, there was a significant association between playing style without ball possession and success in that World Cup. This can provide important information to guide physical preparation and tactical choices to properly prepare national teams for future competitions.

## Data Availability

Publicly available datasets were analyzed in this study. These data can be found here: https://www.fifatrainingcentre.com/.
