# Supplementary material for: The defensive playing style was associated with match-running performance and team success in the FIFA Women's 2023 World Cup
Source: Front Sports Act Living. 2026 Apr 10;8:1693238. doi: 10.3389/fspor.2026.1693238 (PMC13107637; doi:10.3389/fspor.2026.1693238)
Supplement: Supplementary file 1 [file Table1.docx]

**Table S1.** Distribution of match records team and quartile (quartile according to their respective 2023 World Cup final classification) after exclusion criteria.

| Team | Quartile | Match played analyzed |
| --- | --- | --- |
| Spain | 1 | 6 |
| England |  | 6 |
| Sweden |  | 6 |
| Australia |  | 6 |
| Japan |  | 4 |
| Netherlands |  | 4 |
| France |  | 4 |
| Colombia |  | 4 |
| USA | 2 | 3 |
| Nigeria |  | 2 |
| Denmark |  | 4 |
| Morrocco |  | 4 |
| Jamaica |  | 3 |
| Switzerland |  | 4 |
| Norway |  | 3 |
| South Africa |  | 4 |
| Germany | 3 | 3 |
| Brazil |  | 3 |
| Portugal |  | 3 |
| New Zealand |  | 3 |
| Canada |  | 2 |
| Italy |  | 3 |
| China |  | 2 |
| Philippines |  | 2 |
| Zambia | 4 | 2 |
| Ireland |  | 3 |
| Argentina |  | 3 |
| South Korea |  | 3 |
| Haiti |  | 2 |
| Costa Rica |  | 3 |
| Panama |  | 3 |
| Vietnam |  | 3 |

**Table S2.** Descriptive data of phases of play out of ball possession behavior and total distance covered during matches in the FIFA Women's 2023 World Cup per quartile (quartile according to their respective final classification).

| **Variables** | | **Quartiles** | **N** | **Mean** | **Std. Deviation** |
| --- | --- | --- | --- | --- | --- |
|  |  |  |  |  |  |
| Phase of play. out of ball possession (% of time spent in action without ball possession) | High Press (%) | 1 | 40 | 4.08 | 1.91 |
|  |  | 2 | 27 | 2.89 | 1.83 |
|  |  | 3 | 21 | 3.81 | 1.86 |
|  |  | 4 | 22 | 2.77 | 1.90 |
|  | Mid Press (%) | 1 | 40 | 5.88 | 1.92 |
|  |  | 2 | 27 | 5.78 | 2.26 |
|  |  | 3 | 21 | 6.05 | 2.27 |
|  |  | 4 | 22 | 4.73 | 1.78 |
|  | Low Press (%) | 1 | 40 | 0.80 | 0.65 |
|  |  | 2 | 27 | 0.93 | 0.55 |
|  |  | 3 | 21 | 1.14 | 0.48 |
|  |  | 4 | 22 | 1.45 | 0.67 |
|  | High Block (%) | 1 | 40 | 5.95 | 3.23 |
|  |  | 2 | 27 | 4.04 | 2.44 |
|  |  | 3 | 21 | 5.76 | 3.13 |
|  |  | 4 | 22 | 2.86 | 2.10 |
|  | Mid Block (%) | 1 | 40 | 16.18 | 8.41 |
|  |  | 2 | 27 | 23.59 | 10.70 |
|  |  | 3 | 21 | 15.67 | 7.90 |
|  |  | 4 | 22 | 18.41 | 7.71 |
|  | Low Block (%) | 1 | 40 | 12.73 | 8.42 |
|  |  | 2 | 27 | 16.33 | 10.37 |
|  |  | 3 | 21 | 11.38 | 7.02 |
|  |  | 4 | 22 | 22.59 | 13.02 |
|  | Recovery (%) | 1 | 40 | 4.73 | 2.06 |
|  |  | 2 | 27 | 3.70 | 1.77 |
|  |  | 3 | 21 | 4.52 | 2.09 |
|  |  | 4 | 22 | 3.77 | 1.54 |
|  | Defensive Transition (%) | 1 | 40 | 22.85 | 7.07 |
|  |  | 2 | 27 | 18.93 | 6.14 |
|  |  | 3 | 21 | 24.76 | 6.43 |
|  |  | 4 | 22 | 19.14 | 4.82 |
|  | Counter-press (%) | 1 | 40 | 16.30 | 5.03 |
|  |  | 2 | 27 | 13.44 | 3.78 |
|  |  | 3 | 21 | 17.57 | 4.02 |
|  |  | 4 | 22 | 13.91 | 3.34 |
| Defensive Actions (N) | Forced Turnovers (N) | 1 | 40 | 82.78 | 15.15 |
|  |  | 2 | 27 | 84.15 | 12.23 |
|  |  | 3 | 21 | 82.43 | 13.27 |
|  |  | 4 | 22 | 80.32 | 9.34 |
|  | Possession Regained (N) | 1 | 40 | 54.45 | 7.32 |
|  |  | 2 | 27 | 58.56 | 10.60 |
|  |  | 3 | 21 | 57.14 | 9.45 |
|  |  | 4 | 22 | 56.05 | 6.80 |
|  | Interceptions (N) | 1 | 40 | 8.13 | 3.78 |
|  |  | 2 | 27 | 9.00 | 4.14 |
|  |  | 3 | 21 | 8.81 | 2.58 |
|  |  | 4 | 22 | 8.05 | 3.06 |
|  | Tackles (N) | 1 | 40 | 35.45 | 10.53 |
|  |  | 2 | 27 | 37.93 | 11.14 |
|  |  | 3 | 21 | 35.33 | 8.78 |
|  |  | 4 | 22 | 44.59 | 11.30 |
| Defensive Pressure | Total Pressures (N) | 1 | 40 | 200.05 | 63.37 |
|  |  | 2 | 27 | 246.70 | 66.92 |
|  |  | 3 | 21 | 204.62 | 77.24 |
|  |  | 4 | 22 | 271.91 | 75.08 |
|  | Direct Pressures (N) | 1 | 40 | 56.00 | 14.23 |
|  |  | 2 | 27 | 63.89 | 16.84 |
|  |  | 3 | 21 | 56.57 | 16.13 |
|  |  | 4 | 22 | 66.36 | 15.93 |
|  | Avg Pressure Duration (N) | 1 | 40 | 1.46 | 0.16 |
|  |  | 2 | 27 | 1.46 | 0.14 |
|  |  | 3 | 21 | 1.49 | 0.21 |
|  |  | 4 | 22 | 1.48 | 0.11 |
|  | Forced Turnovers (N) | 1 | 40 | 82.78 | 15.15 |
|  |  | 2 | 27 | 84.15 | 12.23 |
|  |  | 3 | 21 | 82.43 | 13.27 |
|  |  | 4 | 22 | 80.55 | 9.18 |
|  | Ball Recovery Time (seconds) | 1 | 40 | 8.75 | 3.06 |
|  |  | 2 | 27 | 10.48 | 2.85 |
|  |  | 3 | 21 | 8.45 | 2.94 |
|  |  | 4 | 22 | 11.38 | 2.93 |
|  | Pushing on into Pressing (N) | 1 | 40 | 74.23 | 32.28 |
|  |  | 2 | 27 | 92.89 | 31.09 |
|  |  | 3 | 21 | 79.00 | 37.42 |
|  |  | 4 | 22 | 104.05 | 41.00 |
|  | Pushing on (N) | 1 | 40 | 164.17 | 65.92 |
|  |  | 2 | 27 | 211.70 | 66.22 |
|  |  | 3 | 21 | 179.62 | 77.98 |
|  |  | 4 | 22 | 237.73 | 82.99 |
|  | Pressing Direction Inside (N) | 1 | 40 | 31.88 | 10.36 |
|  |  | 2 | 27 | 39.26 | 12.05 |
|  |  | 3 | 21 | 30.67 | 14.24 |
|  |  | 4 | 22 | 41.86 | 15.32 |
|  | Pression Direction Outside (N) | 1 | 40 | 101.38 | 42.26 |
|  |  | 2 | 27 | 130.00 | 41.19 |
|  |  | 3 | 21 | 106.52 | 48.30 |
|  |  | 4 | 22 | 142.95 | 44.23 |
| Contextual variables | Total Distance Covered (km) | 1 | 40 | 108.89 | 5.63 |
|  |  | 2 | 27 | 108.49 | 5.60 |
|  |  | 3 | 21 | 109.77 | 5.36 |
|  |  | 4 | 22 | 105.35 | 5.12 |
|  | High Speed Distance Covered (km) | 1 | 40 | 4.87 | 0.56 |
|  |  | 2 | 27 | 4.76 | 0.63 |
|  |  | 3 | 21 | 5.21 | 0.47 |
|  |  | 4 | 22 | 4.77 | 0.71 |
|  | Possession (%) | 1 | 40 | 49.65 | 12.16 |
|  |  | 2 | 27 | 35.79 | 9.59 |
|  |  | 3 | 21 | 46.68 | 13.21 |
|  |  | 4 | 22 | 33.42 | 10.45 |
